# Dynamic cardiac MRI with high spatiotemporal resolution using accelerated spiral-out and spiral-in/out bSSFP pulse sequences at 1.5 T

**DOI:** 10.1007/s10334-023-01116-9

**Published:** 2023-09-04

**Authors:** Zhixing Wang, Xue Feng, Michael Salerno, Christopher M. Kramer, Craig H. Meyer

**Affiliations:** 1 Department of Biomedical Engineering, University of Virginia, Charlottesville, VA USA; 2 School of Medicine, University Medical Line, Stanford University, Stanford, CA USA; 3 Cardiovascular Division, Department of Medicine, University of Virginia, Charlottesville, VA USA; 4 Department of Radiology and Medical Imaging, University of Virginia, Charlottesville, VA USA

**Keywords:** Dynamic imaging, bSSFP, Spiral trajectory, Low-rank plus sparse

## Abstract

**Objective:**

To develop two spiral-based bSSFP pulse sequences combined with L + S reconstruction for accelerated ungated, free-breathing dynamic cardiac imaging at 1.5 T.

**Materials and methods:**

Tiny golden angle rotated spiral-out and spiral-in/out bSSFP sequences combined with view-sharing (VS), compressed sensing (CS), and low-rank plus sparse (L + S) reconstruction were evaluated and compared via simulation and in vivo dynamic cardiac imaging studies. The proposed methods were then validated against the standard cine, in terms of quantitative image assessment and qualitative quality rating.

**Results:**

The L + S method yielded the least residual artifacts and the best image sharpness among the three methods. Both spiral cine techniques showed clinically diagnostic images (score > 3). Compared to standard cine, there were significant differences in global image quality and edge sharpness for spiral cine techniques, while there was significant difference in image contrast for the spiral-out cine but no significant difference for the spiral-in/out cine. There was good agreement in left ventricular ejection fraction for both the spiral-out cine (− 1.6 $\pm$ 3.1%) and spiral-in/out cine (− 1.5 $\pm$ 2.8%) against standard cine.

**Discussion:**

Compared to the time-consuming standard cine (~ 5 min) which requires ECG-gating and breath-holds, the proposed spiral bSSFP sequences achieved ungated, free-breathing cardiac movies at a similar spatial (1.5 × 1.5 × 8 mm^3^) and temporal resolution (36 ms) per slice for whole heart coverage (10–15 slices) within 45 s, suggesting the clinical potential for improved patient comfort or for imaging patients with arrhythmias or who cannot hold their breath.

**Supplementary Information:**

The online version contains supplementary material available at 10.1007/s10334-023-01116-9.

## Introduction

In clinical practice, cardiac magnetic resonance images are often acquired during a breath-hold using an electrocardiography-gated (ECG-gated) balanced steady-state free precession (bSSFP) pulse sequence with segmented Cartesian readouts [1]. While producing images with high image quality and spatiotemporal resolution, this conventional method is time-consuming, typically taking 5–6 min to cover the whole left ventricle, and may be contaminated by ECG-gating and breathing artifacts. Thus, dynamic imaging that does not rely on cardiac gating or breath-holding may be advantageous for patients with arrhythmias or who have difficulties in holding their breath [2–4].

Compressed sensing (CS) [5] has been widely used to accelerate data acquisition in dynamic imaging. By exploiting extensive spatiotemporal data redundancy, this technique allows highly undersampled MRI data to be reconstructed without loss of information. Moreover, CS can be extended to further increase imaging speed, for example, by combining it with parallel imaging [6, 7] (PI) to exploit joint sparsity across images captured by multiple coils (e.g., *k*-*t* SPARSE-SENSE [8–10]), or by combining it with low-rank matrix completion to enable reconstruction of a matrix with missing entries under low-rank and incoherent conditions (e.g., L&S [11], L + S [12]). To date, the L + S model has been investigated in several applications, including cardiac imaging [12, 13], speech imaging [14], and temperature imaging [15], for its ability to achieve good image quality with high acceleration rates.

Since CS-based methods require a sparse representation, incoherent aliasing artifacts are often generated via random under-sampling of Cartesian *k*-space or the use of non-Cartesian sampling patterns [5]. The achievable incoherence from a 2D Cartesian *k*-space trajectory, however, is relatively low when compared to that from non-Cartesian trajectories. Therefore, non-Cartesian bSSFP sequences, such as radial- or spiral-based trajectories, may improve dynamic cardiac MRI using intrinsic variable density trajectories. Several investigators have demonstrated that radial [10, 16–18] and spiral-out [19–21] sampling patterns inherently generate incoherent aliasing artifacts and can achieve high acceleration capability when combined with advanced acceleration methods, both due to their advantageous time-efficiency and inherent robustness to flow and motion artifacts.

In this work, two optimized spiral-based (spiral-out and spiral-in/out) bSSFP pulse sequences were designed and combined with the reconstruction methods to produce highly accelerated cardiac MRI under ungated free-breathing conditions [22, 23]. A comparison of these combinations was then conducted in normal volunteers. Finally, the proposed methods were validated against a standard ECG-gated breath-hold cine sequence under whole heart coverage short-axis views.

## Methods

### Pulse sequence design

**Fig. 1:**
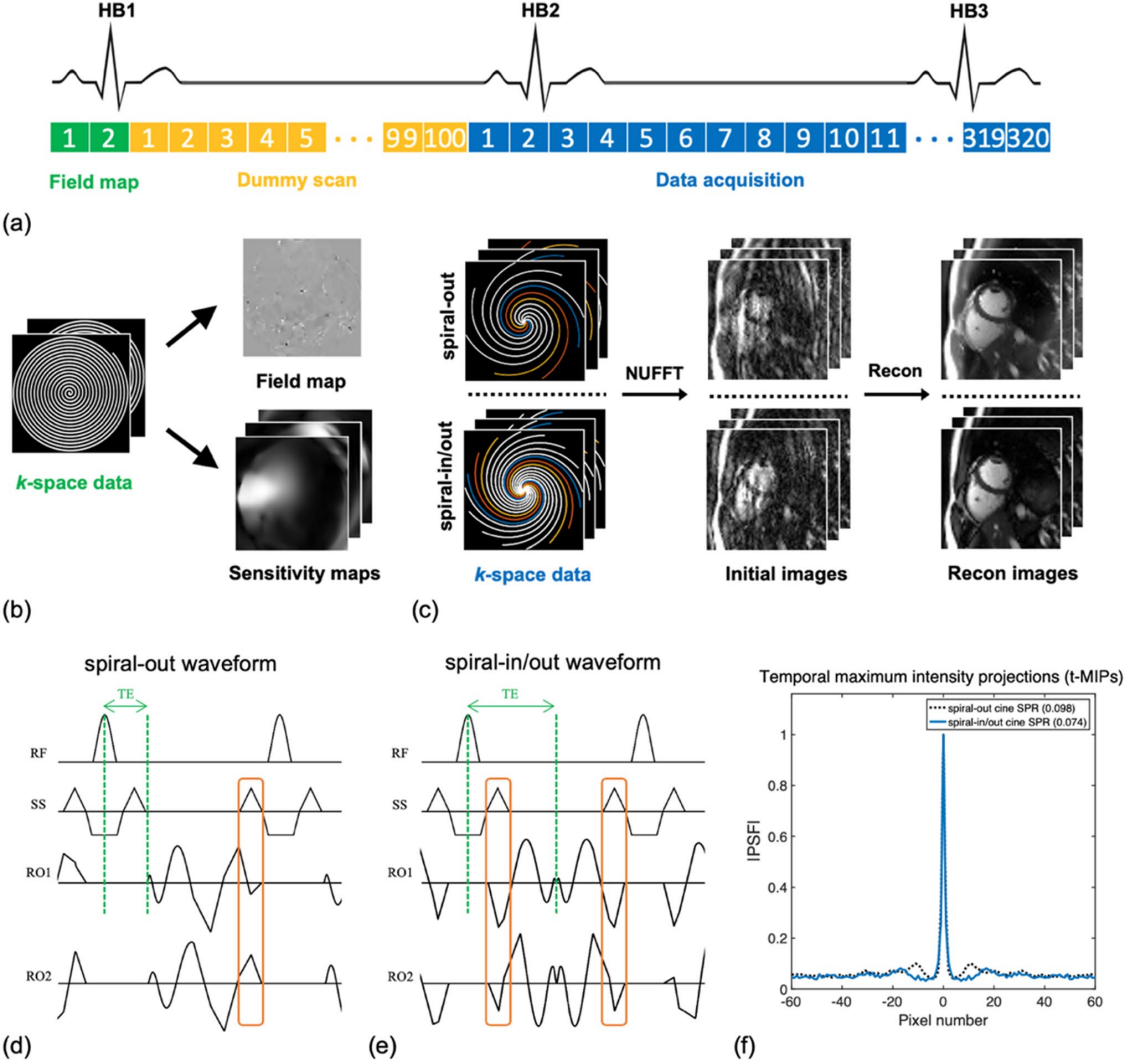
(**a**) Pulse sequence diagram showing the sampling strategy with the field map acquisition, dummy scan, and dynamic data acquisition. (**b**) The field map and sensitivity maps were estimated using the fully sampled center of k-space data collected from the field map acquisition. (**c**) Acquisition and reconstruction pipeline used for dynamic spiral cine imaging. Pulse sequence diagrams within one TR using the spiral-out waveforms (**d**) and spiral-in/out waveforms (**e**). The orange boxes indicate the overlaps between the readout and the slice selection. Note that the spiral-in/out trajectory has a longer acquisition window (ADC: 2.04 ms) than the spiral-out trajectory (ADC: 1.28 ms) for a fixed TR. TE was set to the minimum for the spiral-out cine and to be one half of the TR for the spiral-in/out cine, as indicated by green lines. (**f**) Analysis of PSFs over time for the spiral-out and spiral-in/out readouts. The results show the central 120 pixels of signal intensities across a line over the central region of the t-MIPs (each having 220 × 220 pixels) for both spiral trajectories

Figure 1a shows the timing diagram of dynamic spiral-based cine sequences within a single scan. Two low spatial resolution datasets were acquired before the bSSFP module using spectral-spatial [24] RF water excitation pulses and two identical, single-shot fully sampled spiral-out arms (green boxes) with two TEs ($\Delta TE=1 ms$). A field map with 9 × 9 mm^2^ in-plane resolution was then generated, followed by means of the maximum likelihood algorithm to estimate a linear map $f(x,y)$, using the equation below,

1 $$f\left(x,y\right)={f}_{0}+\alpha x+\beta y,$$

where ${f}_{0}$ is the center frequency, and $\alpha ,\beta$ are the gradients along x and y axis, respectively. Coil sensitivity maps were also calculated from this fully sampled field map dataset using ESPIRiT [25]. Dummy cycles (yellow boxes) with a total number of 100 TRs were then used to approach steady-state magnetization [26]. Immediately after the pre-scan, dynamic data with a total of 320 spiral arms per slice were collected using a tiny golden angle rotation (blue boxes). Interleaved, rotated spiral-out and spiral-in/out readouts were both evaluated in this study.

For spiral-out gradients, we generated a spiral-out arm constrained by the gradient amplitudes and slew rates in a minimum time [27], along with the following rewinder gradients with zeroth and first-order moments nulling via triangular gradients [20], as shown in Fig. 1d. The rewinder gradient was designed to overlap with the next slice-selection prewinder to shorten the minimum TR. The resulting spiral-out readout was 1.28 ms in length. Figure 1e shows the gradients for a spiral-in/out readout using the algorithm described in Ref. 19. Based on the symmetry of the readout (the preceding time-reversed spiral-in arm and the following spiral-out arm), the spiral-in/out prewinder and rewinder waveforms were designed to overlap with the current slice-selection rewinder and the next slice-selection prewinder gradients, respectively. This property enables a shorter TR or a longer spiral readout compared to the spiral-out trajectory in which the overlap is only feasible on one side. Here, we fixed the TR and chose a longer spiral-in/out readout in our experiments. The resulting spiral-in/out readout was 2.04 ms in length.

A variable sampling density spiral (VDS) scheme using dual-density from 1.5 × to 0.3 × Nyquist was designed for the spiral-out trajectories and from 2.3 × to 0.4 × Nyquist for the spiral-in/out trajectories, both containing a total of 64 arms. The radius of the density transition was set to be one third of the *k*-space radius. Among interleaves, tiny golden ratio angle increments were used for both spiral-out and spiral-in/out cines to reduce phase disturbances induced by eddy currents in bSSFP acquisition. Tiny golden angles were defined using the golden ratio $\tau =(1+\surd 5)/2$ and the sequence

2 $${\psi }_{N\_sym}=\frac{\pi }{\tau +N-1},\;\;\; N = \mathrm{1,2},\dots ,$$

for symmetric center-through trajectories such as spiral-in/out [28]. For asymmetric trajectories such as spiral-out, the angle sequence was

3 $${\psi }_{N\_asym}=\frac{2\pi }{\tau +N-1},\;\;\; N = \mathrm{1,2},\dots .$$

Here we used the golden angle of 23.63 $^\circ$ and 47.26 $^\circ$ for the spiral-in/out readout and the spiral-out readout, respectively. Eight sequential arms were combined to reconstruct each cardiac frame with a nominal acceleration ratio of eight. The actual acceleration ratios, after considering the variable dual-density sampling, were ~ 5.3 × and ~ 3.5 × at the center of *k*-space and ~ 26.7 × and ~ 20 × at the outer of *k*-space for spiral-out and spiral-in/out, respectively. The number of slices varies from 10 to 15 depending on the size of the heart. The total acquisition time was 2–3 s per slice in vivo experiments. Other sequence parameters were given in Table 1.

**Table 1:** Sequence parameters for the spiral-out bSSFP cine, the spiral-in/out bSSFP cine, and the standard ECG-gated, breath-hold Cartesian bSSFP cine

							Resolution	
Sequences	FOV (mm^2^)	TR/TE (ms)	FA	GA	Spiral density	#Readouts	Spatial (mm^3^)	Temporal (ms)
Spiral-out cine	330 × 330	4.48/1.05	60°	47.26°	1.5 × –0.3 ×	8	1.50 × 1.50 × 8	36
Spiral-in/out cine	330 × 330	4.44/2.22	60°	23.63°	2.3 × –0.4 ×	8	1.51 × 1.51 × 8	36
Standard breath-hold cine	330 × 260	2.7/1.3	60°	–	–	108	1.50 × 1.50 × 8	40

### Analysis of point spread functions

Point spread functions (PSFs) of spiral-out and spiral-in/out sampling patterns were both computed for each time frame with eight spiral arms per frame. The maximum intensity projection (t-MIP) [29] over the time domain was computed from each series of a total of 80 PSFs. The sidelobe-to-peak ratio (SPR) [5] was then calculated for each trajectory using Eq. [4] as follows:

4 $$SPR= \underset{i\ne j}{\mathrm{max}}\left( \frac{{PSF}_{i,j}}{{PSF}_{i,i}} \right).$$

### System imperfections

In spiral imaging, *k*-space infidelity may cause image blurring or distortion [19, 30, 31]. In this paper, anisotropic gradient delays and eddy current coefficients were calculated for each axis to estimate the actual *k*-space trajectories, for both the spiral-out and spiral-in/out readouts, using a model-based trajectory estimation method [19, 31]. Off-resonance effects may also cause severe banding artifacts in bSSFP data acquisition and image blurring during the spiral readout. In this work, a vendor provided cardiac shim package was used to improve field homogeneity, and time-optimized gradients were designed to achieve a TR of less than 4.5 ms for both spiral bSSFP cine sequences to reduce the banding artifacts. Furthermore, a linear off-resonance correction which was estimated from the acquired low-resolution B_0_ map was performed to compensate for the phase error during the readout [32].

### Image reconstruction

The image reconstruction pipeline is shown in Fig. 1b, c. The L + S reconstruction was implemented using Eq. [5] as follows:

5 $$\underset{L,S}{\mathrm{min}}\frac{1}{2}{\Vert E*(L+S)-d\Vert }_{2}^{2}+{\lambda }_{L}{\Vert L\Vert }_{*}+{\lambda }_{S}{\Vert TS\Vert }_{1},$$

where *E* was a multi-coil encoding operator, including a non-uniform FFT (NUFFT) kernel [33, 34] and the coil sensitivity maps. *L* and *S* were the low-rank and sparse components, respectively. *d* was the undersampled spiral data, and *T* was a temporal total-variation (TTV) operator as the sparsifying transform [12]. The regularization parameters for the low-rank term (${\lambda }_{L}$) and the sparse term (${\lambda }_{S}$) were adjustable parameters to weight these terms (*L* and *S*) relative to data consistency, which were determined by comparing the resulting image quality for a series of testing values. For in vivo datasets, parameters were chosen based on the qualitative assessment of the residual aliasing artifacts and visual temporal blurring (see Supporting Fig. 1).

For comparison, the CS method was implemented by a similar methodology for parameter selection and by directly enforcing the sparsity on the original matrix, using the following Eq. [6]:

6 $$\underset{M}{\mathrm{min}}\frac{1}{2}{\Vert E*M-d\Vert }_{2}^{2}+{\lambda }_{t}{\Vert TM\Vert }_{1},$$

where *M* was the cardiac image series to be reconstructed in *x*–*y*-*t* space, and *T* was the TTV operator used as the only regularization parameter. A view-sharing (VS) reconstruction method was also used for reconstruction comparisons [35].

All computations were performed offline in MATLAB (R2022b software; MathWorks, Natick, MA). The NUFFT operator was utilized for direct spiral image reconstruction. A proximal optimized gradient method (POGM) [36] was used for fast L + S iterative convergence, while a nonlinear iterative conjugate gradient algorithm was used for solving CS optimization problem. The parameters ${\lambda }_{L}$ of 0.05 and ${\lambda }_{S}$ of 0.0005 were determined for the in vivo L + S images, while a ${\lambda }_{S}$ of 0.0006 was chosen to reconstruct the in vivo CS images.

### Simulation

Simulations were performed using the digital cardiac phantom MRXCAT [37]. A series of fully sampled complex-valued cardiac frames were retrospectively undersampled using two aforementioned spiral-based under-sampling patterns (both with eight-arms). Simulation parameters included: image matrix 220 $\times$ 220, 80 frames under free-breathing condition, 16 coils, Gaussian noise with a SNR of 15 db. Image reconstruction was performed using multi-coil CS, and L + S with regularization parameters selected by evaluating the normalized root mean square error (NRMSE) and structural similarity index (SSIM) [38]. NRMSE and SSIM values were then calculated for both of these two spiral trajectories along with reconstruction methods, with the fully sampled image as a reference.

### In vivo* experiments*

All in vivo studies were performed on a 1.5 T scanner (MAGNETOM Avanto, Siemens Healthcare, Erlangen, Germany) with a 32-channel anterior–posterior surface coil array. Seven healthy volunteers (five males and two females) participated in this work with written informed consent prior to the experiment. Whole-heart coverage short-axis views were imaged by these two spiral cine sequences under an ungated, free-breathing condition, and by the standard cine technique during several breath-holds as a reference. For each set of experiments, the three pulse sequences were run consecutively at the same image plane with the same cardiac shimming settings. To be noted, the standard cine was performed using a retrospective ECG-gated bSSFP Cartesian sequence. FOV was set to 330 × 260 mm^2^, with a 40 ms temporal resolution and 1.50 × 1.50 × 8 mm^3^ spatial resolution. Vendor-provided GRAPPA was used for two-fold acceleration, resulting in a total scan time (also referred to as breath-hold time) of 10–12 s per slice. A set of 25 cardiac frames were reconstructed and interpolated based on the ECG signal for each slice. Other sequence parameters can be found in Table 1.

### Image assessment

A blind rating of the diagnostic quality of cine datasets (*n* = 7) was performed by two cardiologists, with a 1 (nondiagnostic)–5 (excellent) scale. Differences in qualitative image quality ratings for dynamic spiral bSSFP cines along with image reconstruction methods were analyzed, followed by pairwise comparisons using the Wilcoxon signed-rank test. Furthermore, to validate the proposed two spiral cine sequences with the L + S reconstruction method against the standard breath-hold Cartesian cine sequence, short-axis movies from all the subjects were then rated and compared, as well.

Measurement of signal-to-noise ratio (SNR) and contrast-to-noise ratio (CNR) in images reconstructed using CS-based methods is not straightforward because of the uneven distribution of noise. Thus, a quantitative calculation of image contrast between the blood pool and the myocardium was performed, and a method similar to that described in Ref 17 and 21 was used to measure the average blood pool and myocardial signal intensity in the end-diastolic midventricular image for each subject. The maximum gradient of signal intensities across the border of the left ventricular (LV) septum was also measured to calculate the edge sharpness (mm^−1^) in every cardiac phase of the midventricular orientation. Differences in image contrast and mean edge sharpness for the two spiral cine techniques with L + S reconstruction and the standard cine sequence were analyzed, and pairwise comparisons were then implemented via the Wilcoxon signed-rank test.

### Ejection fraction calculation

The left ventricular end-diastolic volume (LV EDV) and end-systolic volume (LV ESV) were measured, and the left ventricular ejection fraction (LV EF) was then calculated for the dynamic spiral cines and the standard cine. The agreements in LV EF between the proposed methods and the reference were calculated using a Bland–Altman analysis.

## Results

### Analysis of PSFs over time

Figure 1f shows the signal intensities across a line over the central region of the t-MIPs from the spiral-out cine and spiral-in/out cine. For spiral-in/out cine, the maximum aliasing (SPR: 0.074) is lower within a circular region near the PSF peak, compared to that from spiral-out cine (SPR: 0.098). For a fixed TR, a longer spiral-in/out readout shows a higher level of incoherence than the spiral-out readout, which results in improved characteristics for sparse image reconstruction.

### Simulation

**Fig. 2:**
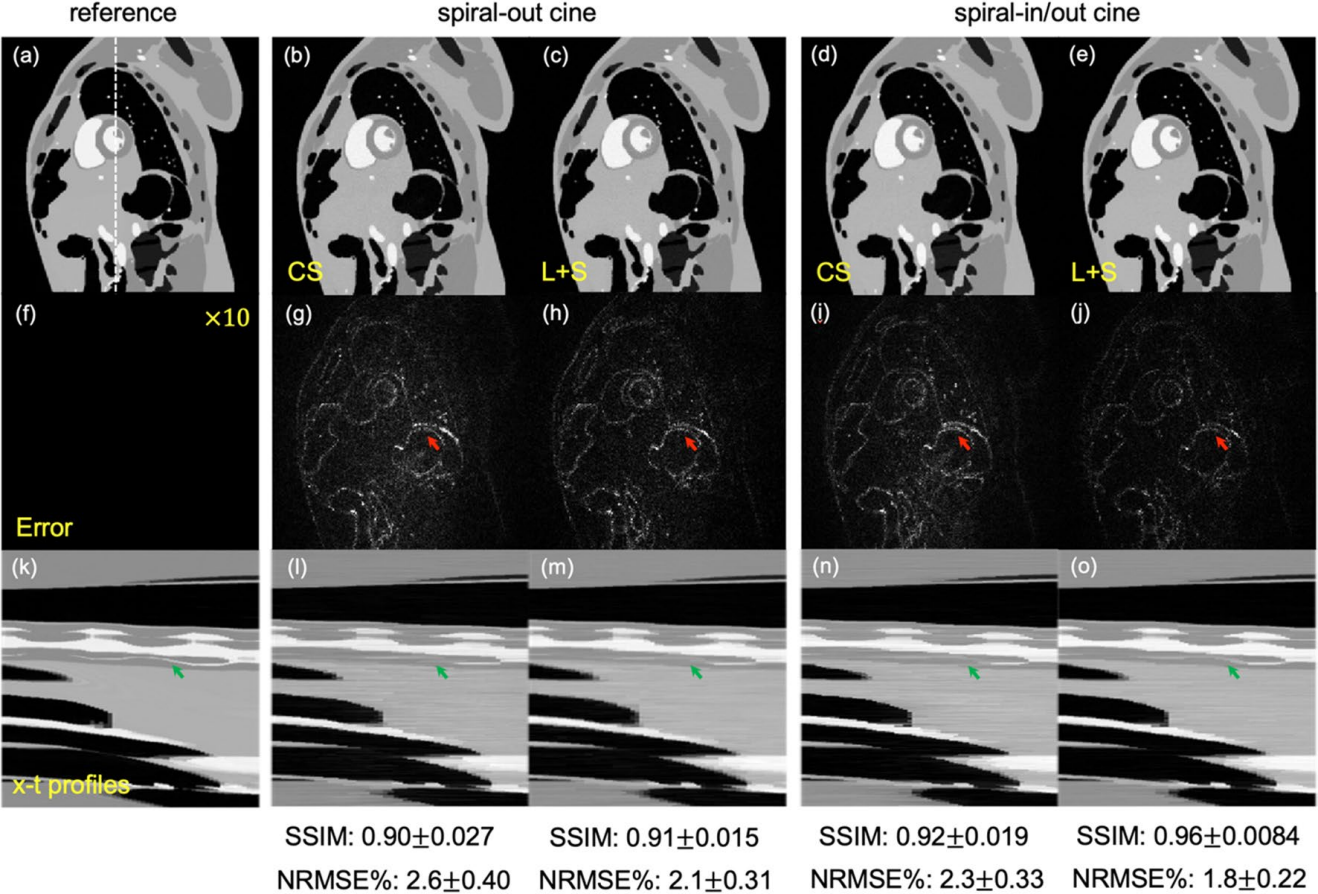
Simulation of a numerical cardiac phantom for the spiral-out cine and the spiral-in/out cine with CS and L + S reconstruction methods (**a**–**e**). Absolute difference images (**f**–**j**) relative to the fully sampled reference (e.g., (**g**) shows the difference between image (**b**) and the reference image (**a**)), and *x*-*t* profiles (**k**–**o**), as well as the corresponding SSIM and NRMSE values (mean $\pm$ standard deviation), are shown for comparison. The error maps are windowed by scaling the image intensity by a factor of ten. Red arrows show the regions with image artifacts, while green arrows show the temporal information

Figure 2 shows the numerical phantom images and the x-t profiles reconstructed from the spiral-out and spiral-in/out readouts using CS and L + S reconstruction methods, and the fully sampled image as the reference. The results indicate that all methods lead to a high SSIM value (> 0.90) and a low NRMSE value (< 3%). Among these methods, the spiral-in/out cine with L + S method has the lowest-intensity image artifacts (red arrows) and maintains the best temporal information (green arrows). The same result can also be found from the in vivo midventricular cardiac images, movies, and the corresponding image ratings (see Supporting Fig. 2, Online Resource 2, and Supporting Fig. 3, respectively).

### In vivo cine images

**Fig. 3:**
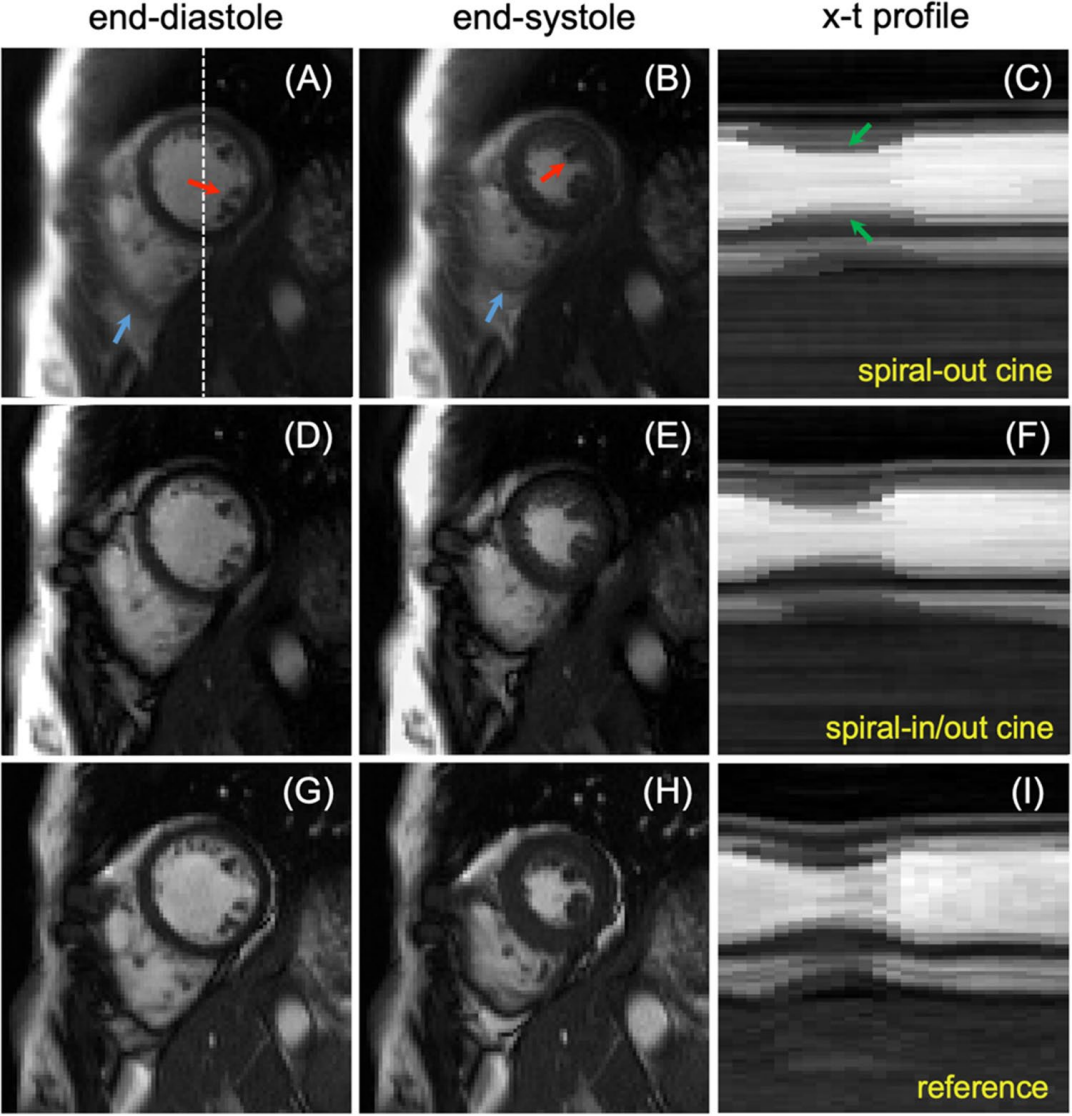
Comparison of reconstructed cardiac frames from a healthy volunteer using free-breathing spiral-out cine (**a**–**c**), free-breathing spiral-in/out cine (**d**–**f**), and standard breath-hold Cartesian cine (**g**–**i**). The spiral images were reconstructed using the L + S method. End-diastolic and end-systolic images are shown in the first and second columns, respectively. The white dashed line represents the location used to derive the *x*-*t* profile. Red arrows point to structures that show fine papillary muscles in ventricles, while green arrows indicate the preserved temporal fidelity. Blue arrows point to structures where both the spiral-in/out cine and standard cine show clear right ventricular endocardial borders while the spiral-out cine does not. The spiral-in/out bSSFP images show image contrast between the blood pool and myocardium closer to that of standard Cartesian cine images than the spiral-out bSSFP images

Figure 3 shows images from one healthy volunteer using spiral-out cine (top) and spiral-in/out cine (middle) with L + S reconstruction, and the standard Cartesian cine (bottom). End-diastolic and end-systolic images are shown in the first and second columns, respectively. Fine papillary muscles (red arrows) are clearly visualized in ventricles in images from the dynamic spiral cines. Green arrows pointing to x-t profiles demonstrate the preserved temporal fidelity of these two spiral cine techniques with a slight difference compared to that of the standard Cartesian cine, potentially due to the through-plane respiratory movement in the dynamic techniques. The corresponding videos can be found in Online Resource 3.

**Fig. 4:**
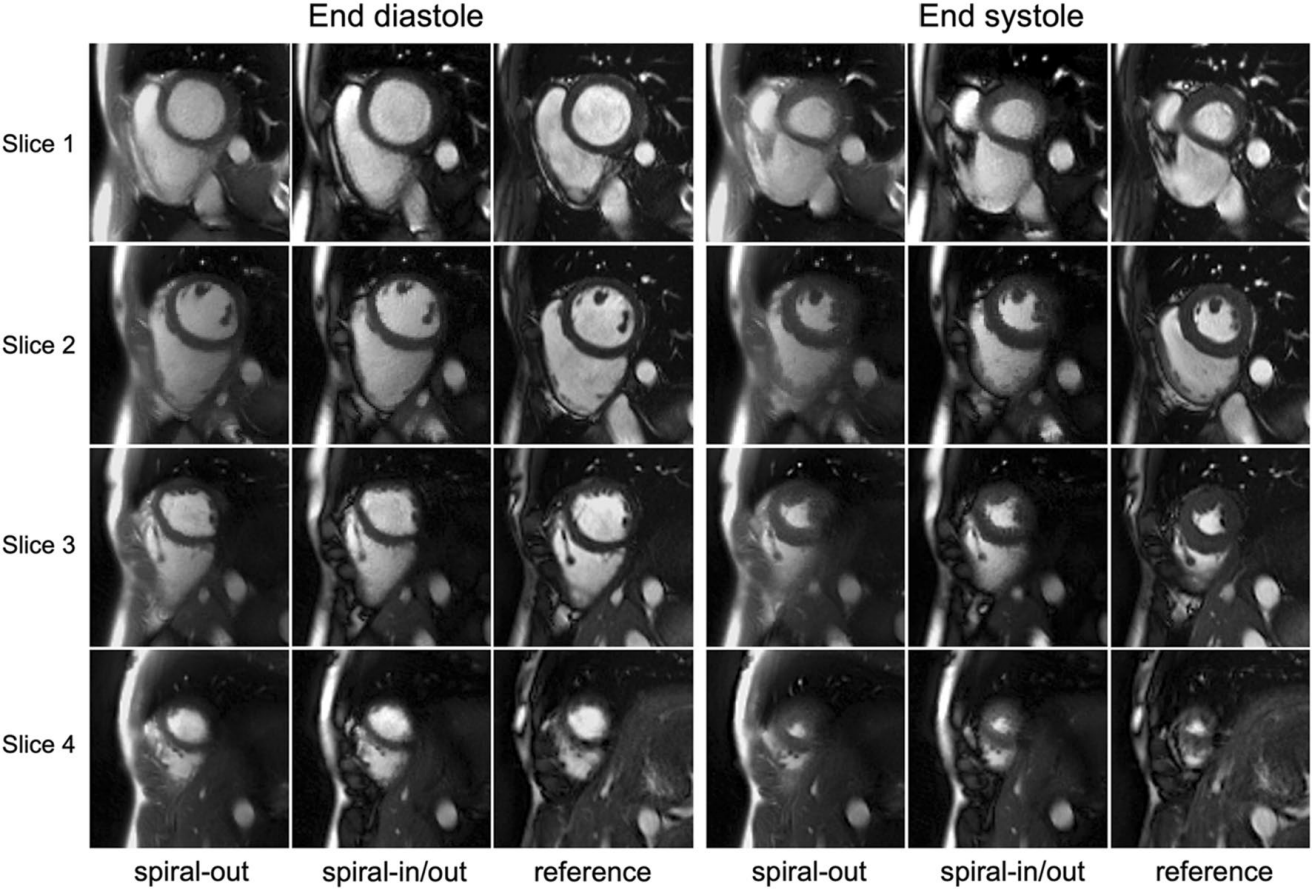
Comparison of short-axis cardiac images from free-breathing spiral-out (left column), free-breathing spiral-in/out (middle column), and standard breath-hold Cartesian cine (right column) in one healthy volunteer. For each method, four slices at end diastole (left) and end systole (right), from apex (bottom) to base (top) are shown

Figure 4 shows representative sets of short-axis cine images from another healthy volunteer, using dynamic spiral-out and spiral-in/out bSSFP sequences with L + S reconstruction, and the breath-hold reference. Both the spiral-based sequences produce overall good diagnostic image quality (rating scores > 3), while the spiral-in/out cine shows a visually better image contrast between the blood pool and the myocardium than the spiral-out cine. The standard breath-hold images (bottom row) show blurring artifacts, potentially because of a poor ECG-trigger signal. The corresponding videos are in Online Resource 4.

### Image assessment

**Fig. 5:**
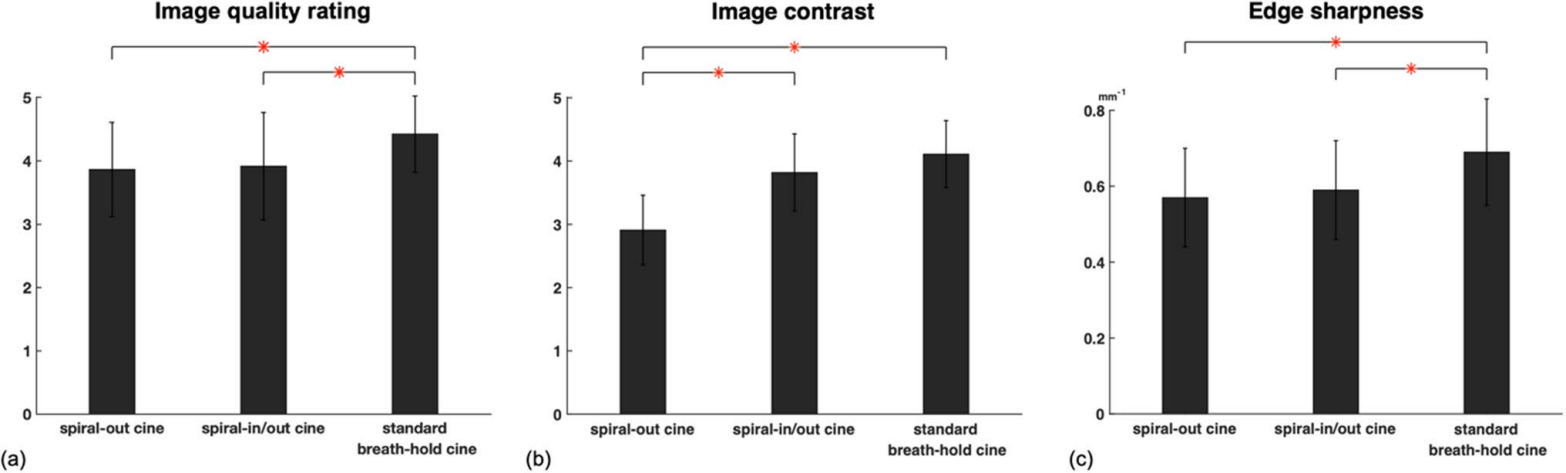
Assessment of image quality of spiral-out cine, spiral-in/out cine, and standard breath-hold Cartesian cine images. (**a**) Subjective assessment of global image quality. (**b**) Quantitative assessment of measured image contrast. (**c**) Quantitative assessment of edge sharpness (unit: mm^−1^). The error bars represent standard deviations. Asterisks indicate a significant difference (*p* < 0.05)

Figure 5a shows that the global image quality of the standard cardiac gated images was 4.4 $\pm$ 0.6, compared to 3.8 $\pm$ 0.8 for spiral-out bSSFP cine with the L + S method and 3.9 $\pm$ 0.7 for spiral-in/out bSSFP cine with the L + S method. The total dataset (*n* = 7) included three short-axis (apex, middle, base) slices per subject. Error bars represent the standard deviations. All cardiac images were diagnostic (score > 3), while the differences in image quality between the spiral-out cine and the standard cine, and between the spiral-in/out cine and the standard cine were both significant (*p* < 0.05). Figure 5b shows the measured image contrast between the blood and myocardium for spiral-out bSSFP cine, spiral-in/out bSSFP cine, and standard Cartesian bSSFP cine. Error bars represent standard deviations. The image contrast was significantly higher (*p* < 0.05) for the standard cine (4.1 ± 0.5) and the spiral-in/out cine (3.8 ± 0.6) than the spiral-out cine (2.9 ± 0.5). Edges (mm^−1^) were sharper in the standard cine (0.69 ± 0.14) than the spiral-out (0.58 ± 0.13) and spiral-in/out cine (0.59 ± 0.13), as shown in Fig. 5c. The difference in edge sharpness between the standard cine and either the spiral-out or spiral-in/out cine was significant (*p* < 0.05), while that was not significant between the spiral-out and spiral-in/out cine.

### Ejection fraction

**Fig. 6:**
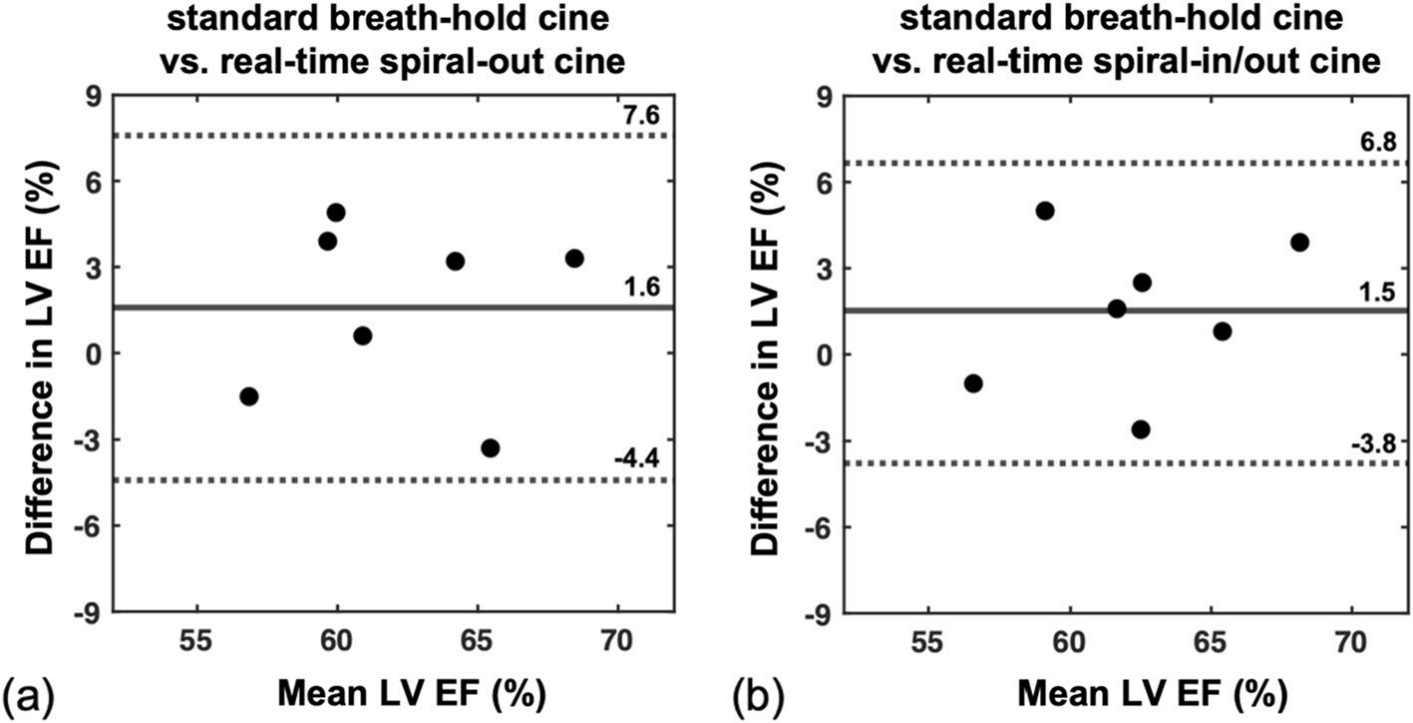
Bland‐Altman plots of LV EF for the subjects with whole‐heart coverage. (**a**) Bland‐Altman plot of EF calculated from the standard Cartesian cine results and the spiral-out cine results. Mean = (Cartesian cine + spiral-out cine)/2, Difference = Cartesian cine—spiral-out cine. (**b**) Bland‐Altman plot of EF calculated from the standard Cartesian cine results and the spiral-in/out cine results. Mean = (Cartesian cine + spiral-in/out cine)/2, Difference = Cartesian cine—spiral-in/out cine. Spiral images were reconstructed using the L + S method

The Bland–Altman plots show the comparison of LV EF between Cartesian cine and the spiral-out cine (Fig. 6a), and between Cartesian cine and the spiral-in/out cine (Fig. 6b). The mean differences in LV EF were − 1.5% and − 1.6% for the spiral-out cine and spiral-in/out cine, respectively, compared to the breath-hold reference. There was no statistical significance among these three methods (*p* > 0.1), establishing the accuracy of estimating LV EF using these two dynamic spiral cine techniques. There was no significant difference in mean LV EDV (*p* > 0.1). However, the mean LV ESV was significantly overestimated (*p* < 0.05) using both of the spiral cine techniques compared to those using the conventional breath-hold cine (see Supporting Table 1).

## Discussion

In this work, we developed and validated both spiral-out and spiral-in/out bSSFP sequences combined with a L + S reconstruction method to achieve highly accelerated, ungated, free-breathing dynamic cine with 1.5 mm in-plane isotropic spatial resolution and 36 ms temporal resolution. The total scan time for whole LV coverage was greatly reduced from approximately 5–6 min with multiple breath-holds to 30–45 s with continuous scanning during free-breathing. The performance of these two sequences for cardiac function was compared to the standard Cartesian cine using protocols with similar spatiotemporal resolution, demonstrating there was good agreement for LV EF calculation between dynamic spiral techniques and the conventional breath-hold Cartesian cine.

Spiral *k*-space scanning has been previously investigated for accelerating ungated, free-breathing cardiac imaging, either with spoiled gradient echo (GRE) sequences [13, 39] or with bSSFP sequences [21]. A GRE strategy may have certain advantages over bSSFP acquisition for its insensitivity to off-resonance effects, allowing a longer spiral readout for a more efficient sampling; however, image contrast of GRE images between the blood pool and the myocardium is inferior compared to that of bSSFP images, which may lead to difficulty and inaccuracy in demarcation of the endocardial surface when calculating LV EF. Steeden et al. [21] used a short uniform density spiral-out (UDS) trajectory with a zeroth order moment rewinder to reduce the off-resonance effects in bSSFP sequences, yet this approach limits its spatial resolution due to a relatively low sampling efficiency within each TR. The unbalanced first moment of the readout gradient may also induce undesirable phase-related in-plane motion and inflow artifacts [20]. In this work, a VDS design instead of the UDS design was used for both spiral-out and spiral-in/out trajectories, which has been demonstrated to suppress under-sampling aliasing artifacts [40, 41] by sampling more at the center but less at the outer portion of *k*-space, thus increasing the level of incoherence that is important for CS-related approaches. Furthermore, the spiral-out trajectory was designed with time-optimized first-order moment nulling gradients, and the spiral-in/out trajectory has intrinsic first-order compensation via symmetry, with each resulting in better flow artifact suppression than a spiral trajectory with zeroth order compensation only.

Compared to the standard cine images, both spiral cine images show some image degradation, primarily due to the slight loss of spatiotemporal resolution and residual aliasing artifacts, as shown in Fig. 5a, c. The reduced spatiotemporal resolution mainly came from (1) a high under-sampling ratio at the outer of *k*-space; and (2) the trade-off between the residual aliasing artifacts and the temporal blurring during image reconstruction process. Comparing these two spiral cine sequences, the refocusing mechanism of the spiral-in/out readout, similar to that in the Cartesian readout, may account for a better image contrast compared to the spiral-out readout, because the signal at the middle of TR has more T_2_-weighted refocused spins (e.g., a darker myocardium). Furthermore, as demonstrated both in Figs. 3 and 4, this refocusing mechanism also produced clear right ventricular endocardial borders in the spiral-in/out cine and standard cine but not in the spiral-out cine, mainly because of a pi phase difference between the blood pool (0 Hz) and fat (− 210 Hz) signal at 1.5 T which results in a signal cancelation. This property could be utilized for improving the border contour of the right ventricle (RV) when calculating RV EF, although this work did not explore it. The magnitude and phase modulation as a function of off-resonance frequency for both the spiral-out and spiral-in/out readout based on current sequence protocols were simulated and can be found in Supporting Fig. 4.

*k*-space misregistration in spiral imaging due to gradient infidelity affects the image quality of spiral images, especially for cardiac scanning with oblique slice orientations. In our implementation, the measured system parameters only need to be calibrated once and can then be used for future scans [19, 30]. B_0_ inhomogeneities can degrade spiral images. In this work, short spiral readouts with acquisition windows of around 2 ms resulted in acceptable blurring artifacts at 1.5 T, which were further reduced by a simple linear off-resonance correction, in case the shimming was not good enough.

The choice of lambda parameters is the key for CS-related image reconstruction methods. Although we have not yet conducted a rigorous search for the optimal values because of the difficulty in quantitatively evaluating the cardiac images in this work, we observed that, for a single dataset, the performance remained unchanged within a small range of the chosen parameters. Thus, among different datasets, we fixed the FOV and spatial/temporal resolution for all seven volunteers, in order to have stable performance of the reconstruction method. In addition, the datasets were normalized to [0 1] before running the reconstruction kernel. We tested the fixed lambdas (${\lambda }_{L}\;{and\; \lambda }_{S})$ on all datasets in this work, finding that the lambdas we chose yielded almost the best overall qualitative image quality, in terms of residual aliasing artifacts and visual temporal blurring. However, these lambda values may still be suboptimal for some in vivo scans because of variations in FOV, SNR, and banding artifacts. More general methods for determining lambdas warrant future investigation, but this is outside the scope of this manuscript.

This work has several limitations. The main limitation is that no patients were studied, so a patient study is needed for clinical validation of the proposed spiral cine methods. For example, patients with arrhythmia, which may produce inferior image quality associated with irregular ECG signals used in conventional Cartesian cine, can be recruited for clinical evaluation. Second, the comparison of LV EF values among methods validated that these two dynamic spiral cine sequences achieved good agreement in the accuracy of LV EF quantification, but a larger study could provide a more reliable analysis of functional parameter quantification. Third, end-diastolic and end-systolic frames from spiral cine scans without ECG-gating may not be matched to the same time points from the standard ECG-gated cine scans. The frame mismatch problem becomes more severe when imaging subjects with variable breathing patterns, although in general, a consistent breathing pattern induced through-plane motion, assuming that all short-axis slices were sampled during a similar respiratory cycle, seems to have an acceptable effect on the LV EF quantitative measurements when summing up the total volume across the whole left ventricle. One solution to minimize the discrepancy in respiratory motion is to perform dynamic cine imaging with 3–4 s short breath-holds per short-axis slice, or to combine this method with a simultaneous multi-slice technique for whole left ventricular coverage within a single breath-hold. Another potential direction is to use a cardiac motion- and respiratory motion-resolved multi-dimensional imaging technique, but this would result in the sacrifice of much-increased total scan time [13, 42, 43]. Fourth, the computational cost for offline image reconstruction in MATLAB is currently around 2–3 min. Optimizing the code and implementing the pipeline in C +  + may enable real-time image reconstruction on commercial scanners.

## Conclusions

Two spiral-based (spiral-out, spiral-in/out) bSSFP sequences with low-rank plus sparse image reconstruction were presented for highly accelerated dynamic cine to evaluate the cardiac function. These methods demonstrated cardiac cine imaging without ECG-gating during free breathing and with diagnostic image quality in normal volunteers, suggesting the potential for clinical dynamic cardiac MR imaging.

### Supplementary Information

Below is the link to the electronic supplementary material.

Supplementary file1 (PDF 397 KB)

Supplementary file2 (MP4 924 KB)

Supplementary file3 (MP4 1832 KB)

Supplementary file4 (MP4 1394 KB)

## Data Availability

Data presented in this study are available upon reasonable request.
