# Supplementary material for: Dynamic cardiac MRI with high spatiotemporal resolution using accelerated spiral-out and spiral-in/out bSSFP pulse sequences at 1.5 T
Source: MAGMA. 2023 Sep 4;36(6):857–67. doi: 10.1007/s10334-023-01116-9 (PMC10667461; doi:10.1007/s10334-023-01116-9)
Supplement: Supplementary file 1 — Supplementary file1 (PDF 397 KB) [file 10334_2023_1116_MOESM1_ESM.pdf]

SUPPORTING INFORMATION

	spiral-out cine	spiral-in/out cine	standard breath-hold cine
LV EDV (mL)	152 ± 35	154 ± 38	156 ± 31
LV ESV (mL)	57 ± 17*	58 ± 19**	53 ± 14*,**
LV EF (%)	60 ± 6	61 ± 7	63 ± 5

Supporting Table 1. Comparison of the averaged LV EDV, LV ESV, and LV EF among spiral-out cine, spiral-in/out cine, and the standard breath-hold cine. ± means standard deviation. * and ** mean the matched pairs are statistically significant (p < 0.05).

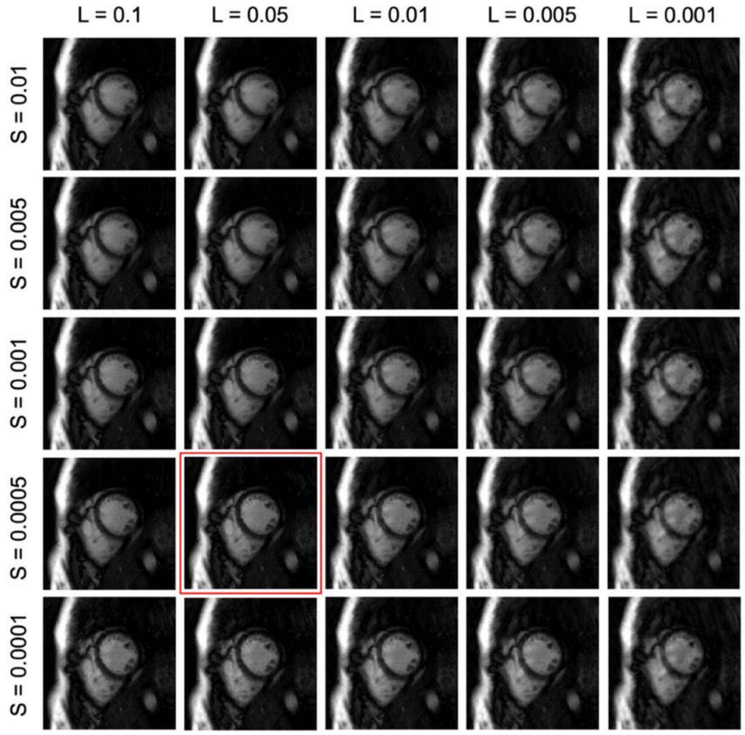

Supporting Figure 1. Reconstructed images of a given cardiac frame using different values of λ_L and λ_S. The image quality of resulting images is sensitive to the parameter selection, especially when selecting the λ_L parameter. At λ_L = 0.05 and λ_S = 0.0005 (red box), the image has the lowest aliasing and temporal blurring artifacts.

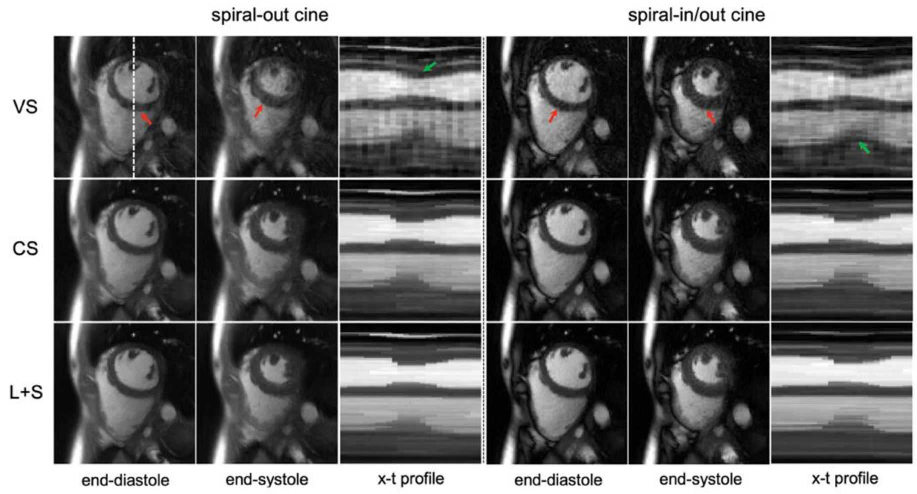

Supporting Figure 2. Comparison of reconstructed end-diastolic and end-systolic frames from a midventricular short-axis view, using the spiral-out (left) and the spiral-in/out (right) bSSFP sequences with VS (top row), CS (middle row), and L+S (bottom row) reconstruction methods. Images were acquired under ungated, free-breathing conditions. The white dashed line represents the location used to derive the x-t profiles. Red arrows point to the structures that show image artifacts, while green arrows show temporal details. The L+S method performs best in terms of artifacts and temporal details.

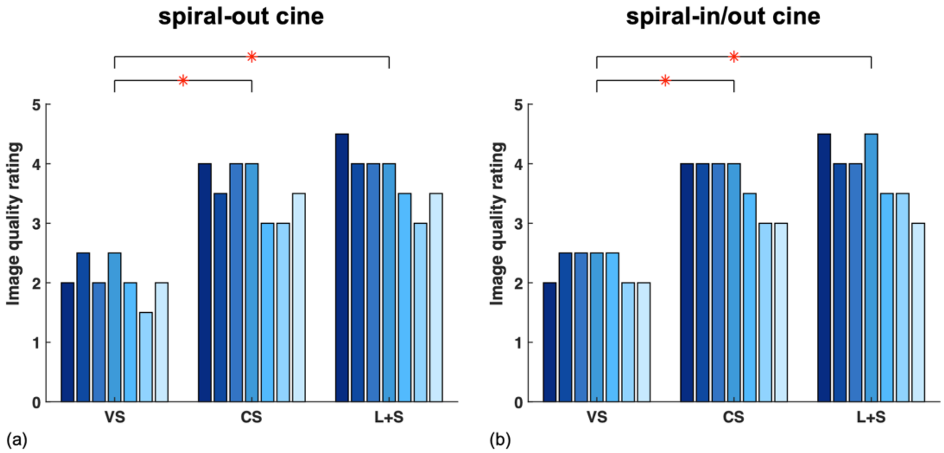

Supporting Figure 3. Image quality ratings among all subjects (n = 7). The bar plot shows the scores for images from the spiral-out cine (a) and from the spiral-in/out cine (b) using VS, CS, and L+S. All scores were graded in a blinded fashion by two cardiologists, each scored from 1 to 5 (worst to best). Asterisks indicate a significant difference ($p < 0.05$).

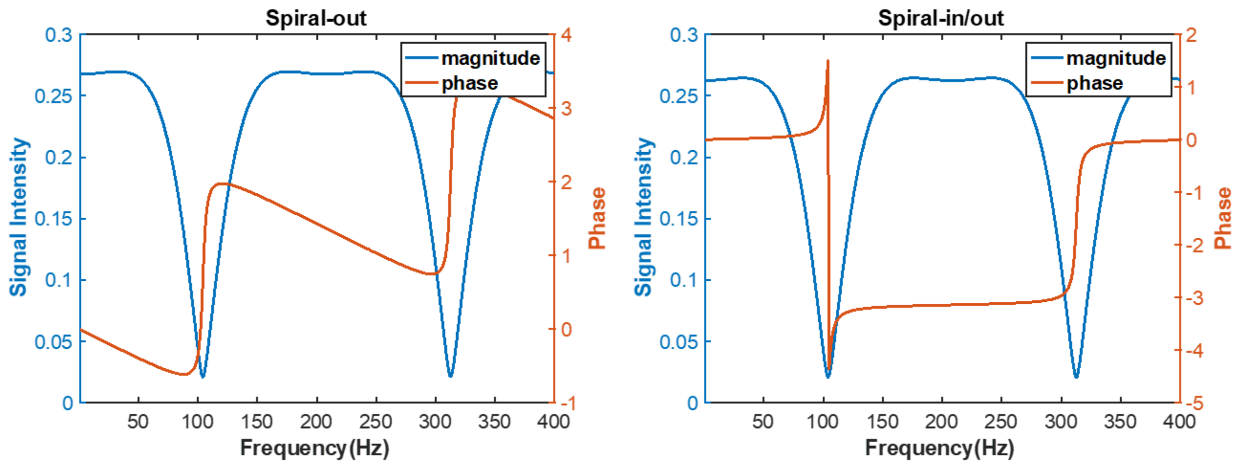

Supporting Figure 4. The magnitude (blue line) and phase (red line) modulation as a function of off-resonance frequency for the spiral-out (left) and spiral-in/out (right) readout at 1.5 T. The current sequence protocols were used for simulation. Note that when comparing between these two spiral readouts, there was a large difference in the phase modulation but no difference in the magnitude modulation. For example, both the water signal (0 Hz) in the spiral-out and spiral-in/out cine has 0 radian phase, while the fat signal (210 Hz) has a nearly +1.3 radian phase in the spiral-out cine but an almost -pi radian phase in the spiral-in/out cine.
